# The influence of an animal's social significance on veterinary antimicrobial use–A hierarchy of care

**DOI:** 10.3389/fvets.2025.1713787

**Published:** 2025-11-28

**Authors:** Gabriela Olmos Antillón, Isabel Blanco-Penedo, Rita Albernaz-Gonçalves, Jo Hockenhull, Maria José Hötzel

**Affiliations:** 1 Department of Clinical Sciences, Veterinary Epidemiology Unit, Swedish University of Agricultural Sciences, Uppsala, Sweden; 2 Department of Animal Science, University of Lleida, Lleida, Spain; 3 Instituto Federal Catarinense, Campus Santa Rosa do Sul, Santa Rosa do Sul, SC, Brazil; 4 Animal Welfare and Behaviour Group, University of Bristol Veterinary School, Langford, United Kingdom; 5 Laboratório de Etologia Aplicada e Bem-Estar Animal, Departamento de Zootecnia e Desenvolvimento Rural, Universidade Federal de Santa Catarina, Florianópolis, Brazil

**Keywords:** social practice theory, meanings, antimicrobial stewardship, animal welfare, ethics

## Abstract

Antimicrobial resistance (AMR) is a pressing global challenge, and veterinary antimicrobial use is a central focus of regulatory and professional scrutiny. While international policy increasingly requires detailed reporting of veterinary AMU, the social dynamics shaping how and why antimicrobials are prescribed, used, and followed up remain poorly understood. This study applies Social Practice Theory, to explore how AMU is enacted across two contrasting species (dairy cattle and dogs) in three countries with differing AMU profiles: Brazil, Spain, and Sweden. The analysis combined policy and guideline review with 187 semi-structured interviews with veterinary specialists, clinicians, farmers, veterinary students, and dog tutors. Interviews examined how common health problems are observed, diagnosed, treated, and followed up, and how participants defined “optimal antimicrobial use” in their contexts. Our reflexive thematic analysis identified five interconnected themes: (1) species-based hierarchies of care, where the perceived value of animals structured AMU tolerance and diagnostic rigor; (2) imagined animal needs, shaped by assumptions of caretaker expectations or legal boundaries; (3) blurred professional roles, with diagnosis and treatment often shared among farmers, technicians, and suppliers; (4) fragile follow-up practices, where “no news” was commonly taken as treatment success and monitoring systems reduced stewardship to counting doses; and (5) entrenched treatment-first logics, in which antimicrobials became the default response, reinforced by professional habits, regulatory scripts, and cultural valuation of animals. This study highlights the importance of recognizing how animals‘ social significance underpins veterinary practices and AMU decisions. Integrating social theory with ethical considerations provides a more nuanced understanding of veterinary practice and antimicrobial stewardship. By foregrounding species-based hierarchies of care, the research demonstrates how animals' social meanings shape antimicrobial decisions, with implications for animal welfare and public health.

## 1 Introduction

Antimicrobial resistance (AMR) represents a critical threat to global health, food security, and sustainable development (1). As antimicrobials lose their effectiveness, the consequences affect both human and animal populations, compromising our ability to manage infections. The veterinary sector plays a pivotal role in this crisis, with widespread calls to reduce antimicrobial use in animals across all domains of care (2).

Numerous international and national initiatives have emerged to address antimicrobial use in veterinary medicine (3–6). However, these efforts often emphasize top-down regulation and awareness campaigns, while overlooking the complex, everyday realities in which veterinary practices are embedded. What remains underexplored is how contextual factors, such as cultural values, professional roles, and societal perceptions of animals influence antimicrobial decisions on the ground. Policies that ignore these contextual elements risk being ineffective or even counterproductive.

This gap in understanding highlights the need for theoretical frameworks capable of capturing the social, material, and ethical dimensions of antimicrobial use. Social practice theory (7, 8) offers such a lens. It moves the focus away from individuals as decision-makers and, instead, examines how antimicrobial use emerges through the interaction of competencies (skills and know-how), materials (infrastructure, tools, and regulations), and meanings (social and emotional significance assigned to practices and species). From this perspective, diagnosing, prescribing and following up treatment success are not simply technical acts–they are socially constructed practices influenced by broader structures and values.

One of the most underexamined dimensions in this framework is the role of meanings: how the animals' perceived social value shapes the care they receive and, by extension, the decisions around antimicrobial use. The contrast between species, such as those used for production and companionship, often reflects deeply embedded assumptions about utility, intimacy, and value, assumptions that are rarely made explicit in antimicrobial use guidelines or stewardship programs. Furthermore, animals also belong to different settings, and the conditions under which they are kept differ substantially due to geography, governance, human resources, health systems availability, production systems, culture and prevailing social norms (9). For example, Sweden is among the countries with the lowest reports of antimicrobial use and AMR in veterinary medicine (10). Spain, in comparison, is a country with high antimicrobial use within the context of EU policies and regulations that include an antimicrobial use reduction strategy shared with Sweden (11). Finally, Brazil is among the top 10 global consumers of antimicrobials for animal use (12) and allows the use of some antimicrobials as growth promoters, contrary to EU policies banning their use since 2006.

In this study, we explored how these meanings operate within and across different national and species contexts. By comparing veterinary antimicrobial use practices involving dogs and dairy cattle in Brazil, Spain, and Sweden, we aim to uncover how the social significance of animals contributes to the formation of antimicrobial practices. This comparative perspective provides new insights into antimicrobial use's ethical and cultural foundations and contributes critically to the design of more grounded, context-sensitive policies.

## 2 Materials and methods

### 2.1 Study design

This study was conducted within the “Antimicrobial Use Veterinary Practices” (AMUVP) project, granted by the Swedish Research Council for Sustainable Development, also known as Formas, with Grant No 2019-00324, a project that spanned from 2020 to 2025. We used an exploratory qualitative design framed by social practice theory. Fieldwork was conducted in Sweden, Spain, and Brazil, countries selected for their contrasting antimicrobial use profiles in veterinary medicine.

### 2.2 Theoretical framework

**Figure 1:**
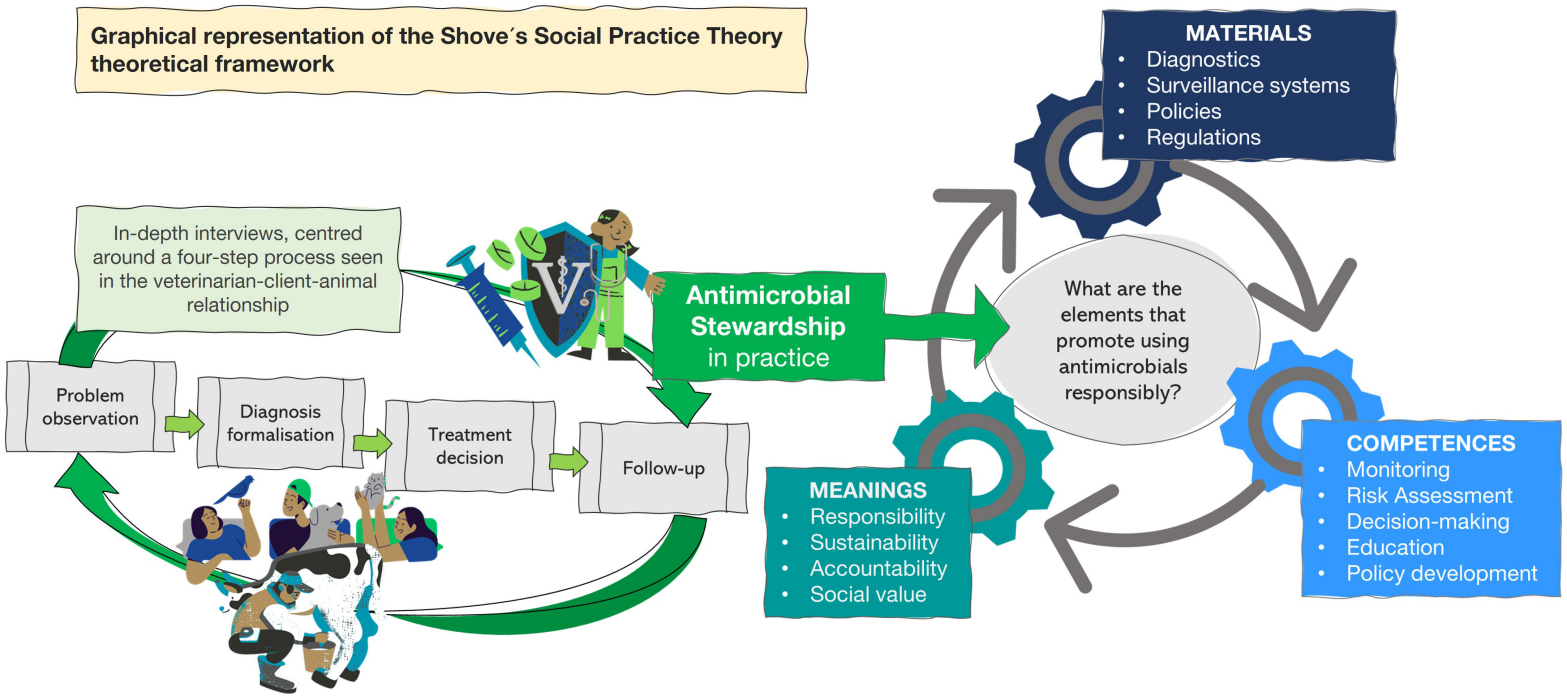
Graphical representation of veterinary antimicrobial use as social practice. The figure frames antimicrobial stewardship (a coherent set of actions toward optimal antimicrobial use) in veterinary medicine as a social practice (8). This veterinary care is organized by elements roughly grouped in *meanings* attached to animals, the *materials* available (diagnostics, surveillance, policy), and *competencies* (monitoring, decision-making). Anchored in this theoretical framework, we map how those elements shape the vet–client–animal process (problem observation, diagnosis, treatment, and follow-up), showing antimicrobial use as a lived practice.

Our analysis was informed by social practice theory (7, 8), which conceives practices as interdependent constellations (see Figure 1) of materials (diagnostics, infrastructures, regulations), competences (veterinary knowledge, diagnostic reasoning, decision-making skills), and meanings (the social and emotional value attributed to animals). While all three elements were considered, this paper emphasizes meanings to examine how the social significance of species shaped veterinary antimicrobial use, producing what we conceptualized as a “hierarchy of care.”

### 2.3 Phase 1—Characterization of legal framework of veterinary practice at the national and regional level

In the first phase, we critically appraised national action plans, veterinary guidelines, and any other framework or legislation relevant to how the veterinary profession is regulated and antimicrobial use is framed within each country or region. Documents analyzed included the EU Veterinary Medicines Directive 2019/6 (11), Spanish national surveillance frameworks and veterinary prescribing regulations (13–20), Brazilian national programmes and veterinary profession regulations (21–30), and Swedish national veterinary guidelines (31–40). This phase provided the structural context for understanding how veterinary practices are framed within governance and regulation. Although this was the project's first phase, close monitoring of new adjustments in these frameworks was maintained across countries for the project's duration.

### 2.4 Phase 2—Veterinary specialists' practice characterization

The second phase consisted of in-depth interviews with specialist veterinarians in dairy cattle, companion animal practice, professors in these areas at key veterinary universities within each country, and veterinarians working at government agencies. In total, 25 semi-structured interviews (see Table 1) were conducted across the three countries. This group of participants were purposely selected based on their expertise in four key clinical scenarios with high antimicrobial use relevance: *E. coli* mastitis and metritis in cattle. In dogs, pyoderma and pyometra. These conditions represent therapeutic and preventive antimicrobial use, often involving a decision between no antimicrobial treatment, first-vs. second-line antimicrobial options, and laboratory-based vs. empirical diagnosis. This focus enables discussion grounded in participants' real experiences rather than generalized accounts of treatment practices. Although including more or different cases could have offered further insights, focusing only on two scenarios per species across countries allowed for a deeper analysis of the breadth of coverage. Furthermore, interviews explored the observation of the problem in a given animal, the formalization of the diagnosis, treatment decisions, and the follow-up of a case until its recovery (see Figure 1). We equally explored perceptions of “optimal” antimicrobial use in general terms.

**Table 1:** Number of participants in in-depth interviews (*n* = 187) done at two study phases across three countries.

**Methodology phase**	**Type of participant**	**Brazil (*n* = 76)**	**Spain (*n* = 66)**	**Sweden (*n* = 45)**
Phase 2 in-depth interviews with veterinary specialists (*n* = 25)	Dairy cattle medicine specialist (*n* = 12)	7	2	3
	Small animal medicine specialist (*n* = 6)	2	2	2
	^*^Other type of specialist (*n* = 7)	2	4	1
Phase 3 in-depth interviews with the broader group of stakeholders (*n* = 162)	Final year veterinary students (*n* = 39)	10	18	11
		Veterinary practitioners dealing daily with clinical conditions of dogs or dairy cattle (*n* = 66)	26	29	11
	Dairy farmer (*n* = 28)	16	6	6
	Dog tutors (*n* = 29)	13	5	11

^*^Other veterinary specialists include veterinarians with clinical specializations (e.g., dermatology, udder health, or reproduction in small or large animals) who worked in regional or university laboratories, governmental agencies, or managerial roles related to veterinary regulation or the use of veterinary drugs within the country.

### 2.5 Phase 3—Everyday veterinary practice characterization

The third phase expanded the interview sample to include a wider group of stakeholders engaged in daily antimicrobial use practices. This included interviews with veterinary practitioners working with dogs or dairy cattle (exclusively or in mixed practice), dog tutors, dairy farmers, and future veterinary professionals (final-year veterinary students). In total, 162 semi-structured interviews (see Table 1) were conducted in this phase across the three countries. As with the veterinary specialists, the in-depth interviews explored the daily experiences and protocols in recognizing clinical problems, same as those elicited in Phase 1, diagnostic routines, treatment decision-making, follow-up practices, and perceptions of responsibility for antimicrobial stewardship (see Figure 1). When interviewing veterinary students, we also included questions that brought out perspectives around university training and real-world constraints.

### 2.6 Participant recruitment

Participants were recruited using a non-probabilistic snowball sampling method, beginning with identifying veterinary specialists (Phase 2) through the interviewers' established professional networks. These initial participants then identified further informants (Phase 3). This method was chosen as it is particularly suited to difficult-to-reach groups, such as practicing veterinarians, farmers, and tutors. The social practice theory guided our study, and the data analysis was done as a reflexive thematic analysis. With this point of departure, the concept of “sample size” is not applied in the same way as in quantitative approaches. In other words, reflexive thematic analysis does not treat adequacy as reaching a predetermined number of participants or a “saturation” point. Instead, adequacy is evaluated by the richness and complexity of the data, the diversity of participant perspectives within the subject explored, and the researcher's reflexive engagement with the material. Specifically, we aimed for the data to provide sufficient depth and breadth to construct meaningful patterns of shared meaning concerning the research question (41–44). Our sample encompassed a plurality of production contexts, species, and geographies, ensuring a wide range of experiences. Most participants held at least a university or professional degree, with farmers varying from vocational agricultural training to higher education. Veterinary students were in their final year, and practitioners ranged from early-career to senior professionals (ages 23–65). Thus, data collection continued until we judged that sufficient informational power had been achieved to address the project's analytic aims. Differences in sample size between countries and participant categories reflect contextual factors such as the number of practicing professionals, farm and clinic density, and accessibility of certain respondent groups. This variation aligns with the study's exploratory, qualitative design, which sought depth and diversity rather than representativeness. Because the snowball sampling strategy is built on existing professional networks, the potential for selection bias cannot be fully excluded. We acknowledge this as a limitation of the study.

### 2.7 Interview procedures and guide

Interviews were semi-structured and based on a shared umbrella guide tailored to each stakeholder group. Topics included:

1. Everyday care routines and animal-human relationships (dog tutors/farmers).
2. Problem observation, diagnostic and treatment procedures for the explored clinical scenarios.
3. Perceptions of antimicrobials, antimicrobial resistance, and “optimal use.”
4. Views on regulation, professional responsibility, and follow-up.

Interviews were conducted face-to-face, by phone, or via online platforms, depending on participant preference and COVID-19 restrictions. They lasted between 20 and 120 min and were conducted in Spanish, Galician, Catalan, Brazilian-Portuguese, Swedish, or English, according to participant choice. GOA, RAG, and IBP, all trained in qualitative interviewing, conducted the interviews.

### 2.8 Data analysis

All interviews across phase 1 and 2 (*n* = 187) were audio-recorded, transcribed in the original language, and pseudonymised. A reflexive thematic analysis (41, 43, 45) was undertaken with all gathered data across the three phases. Coding was open, iterative and collaborative, with transcripts read and coded by team members fluent in each language. Codes were organized according to the three social practice theory elements (materials, competences, meanings). Field notes and post-interview notes (memoing) were also part of the analysis. Coding went beyond the descriptive level of the data to identify the participants' underlying assumptions, ideas and practices around antimicrobial use. To prepare this work, we focused on veterinary practice tensions around the species' social significance (meanings). Themes were compared across stakeholder groups and countries, and triangulated with findings from the legislative review and veterinary specialist interviews. A selection of representative phrases for exemplification of the themes were translated into English when needed by GOA or MJH. The prefixes on their participant number denote the geographical location (BR = Brazil, ES = Spain, SE = Sweden) of the interviewee and the type of stakeholder (S = veterinary specialist, VF = veterinary professional working with dairy cattle, VD = veterinary professional working with dogs, VS = final year veterinary student, DF = dairy farmer and DT = dog tutor).

### 2.9 Ethical statement

The study involved interviews with veterinarians, veterinary students, dairy farmers, and dog tutors in Sweden, Spain, and Brazil. No live animal experimentation was conducted. The Ethics Committee of the Complutense University of Madrid (UCM) registered and approved the study under reference CE_20210715-5_SAL in Spain. At the same time, the study was registered and approved in Brazil by the Ethics Committee on Human Research of the Federal University of Santa Catarina (CEPSH/UFSC) under decision no. 4.567.386. In Sweden, in consultation with the Ethics and Legal Department at the Swedish University of Agricultural Sciences (SLU) and in agreement with the Swedish Ethical Authority, it was determined that the study did not require a special permit according to Swedish law (SFS 2003:460).

In all three countries, participation was voluntary, informed consent was obtained from all interviewees, and confidentiality was guaranteed through pseudo-anonymisation procedures. This ensured that conversations (i.e., responses) and any herd or animal registry were kept safe, and no sensitive personal information was collected. This strictly adheres to the code of conduct set out by the Swedish Research Council (2017). No financial incentives were provided to participants.

In compliance with the General Data Protection Regulation (GDPR, EU 2016/679), all data were processed and stored securely by research staff directly involved in the project. Identifying information was removed before analysis, and only anonymised data were used in publications and dissemination.

### 2.10 Positionality statement

All authors have professional veterinary or animal sciences training in Latin America and Europe. GOAis a veterinarian and mixed methods applied researcher from Mexico who was trained in social science in the UK, and has lived and worked in several countries, including those under the scope of this study. These experiences have shaped her sensitivity to cultural and institutional contrasts in veterinary practice and cultures. IBP is an associate professor in animal welfare and organic farming systems at the University of Lleida (UdL) and Senior Lecturer Adjunct at SLU, Sweden. RAG is a Brazilian veterinarian and professor of animal husbandry with expertise in applied qualitative methodology and a research focus on antimicrobial use in production animals. MJH is a veterinarian trained in qualitative methods and a professor of animal welfare, animal husbandry, and ethics, which grounds her research experience in human-animal relationships and farm practice. JH is a UK-based researcher, specializing in behavior change and qualitative research on veterinary and animal welfare practices.

Together, our positionalities span veterinary medicine, animal science, and social science, with diverse cultural roots. We share a professional commitment to advancing antimicrobial stewardship and animal welfare. However, we recognize that our interpretations are shaped by our training within veterinary and academic institutions, life experiences, and direct observation and participation in caring for animals. This foregrounds specific professional logics while giving visibility to lay perspectives. We sought to engage reflexively and collaboratively throughout the analytic process, being aware of how our backgrounds and commitments influenced the patterns of meaning constructed in the results.

## 3 Results

Guided by Shove's Social Practice Theory (7, 8), and with particular emphasis on meanings, our reflexive thematic analysis constructed a set of interconnected themes that characterize how antimicrobial use is enacted in veterinary practice. Across participants' accounts, antimicrobial use was described not as a purely technical act governed by clinical guidelines, but as a practice shaped by the social significance attributed to different animals, by veterinarians' and caretakers' assumptions of what care should entail, and by the diffuse distribution of professional responsibilities. Limited systems of follow-up and record-keeping further marked these practices, along with a cultural tendency toward a treatment-first logic, in which antimicrobial treatment often became the taken-for-granted response to an observed problem. Together, these themes articulate what we interpret as a “hierarchy of care” in veterinary medicine, where the meanings attached to species underpin both tolerance for antimicrobial use and the degree of diagnostic and follow-up rigour.

### 3.1 Differentiating animals by human values

Across contexts, veterinarians, dairy farmers, and dog tutors described how an animal's perceived worth, whether affective or productive, shaped antimicrobial decision-making. High-valued animals, such as dogs or high-yield dairy cows, were seen as cases where failure was unacceptable, prompting stronger, more tailored, or faster interventions

“*When it comes to dogs or cats, there are often much more emotions involved. It feels like you have to try a little harder, whereas with cows you can think more economically*.” SE_S.03

“*She prescribed an antibiotic, a painkiller and a corticosteroid, it was a cocktail of drugs, I said, I'm not going to give this to my 8-month-old dog…she gave me an alternative*.” BR_DT.10

Together, conversations make visible a valuation logic in which livestock are governed by pragmatic limits distinct from those applied to individualized patients like pets. Veterinarians often drew a sharp line between species and situations observed, something observed even among veterinary students

“*If it's the farmer's best cow, then maybe you give it a chance… but I don't think it would be wrong to cull a cow like that….”* SE_VS.03

“*In such a case, I might not have followed up so carefully… the outcome will in any case be that the farmer culls the animal himself.”* SE_VS.07

For companion animals, dog tutors often narrated intensive, multi-step pathways of care. Such scenarios

“*It was breast cancer, she had to have surgery, went through the whole process before the operation, had to do tests, and now she is well*.” BR_DT.09

differed sharply from accounts about cattle

“*The difference I see between the horse and the cow, for example, before the horse dies you try everything until the last minute. The dog tutor is there at the side, it's something very sentimental. As for the bovine, what do I see? …You can't use a glove because the farmer thinks you're being fancy… So you end up giving all sorts of medications without really knowing what the cow actually has.”* BR_VF.03

Among dairy farmers, especially in larger herds (≥100 lactating cows), emotional ties to individuals were said to be replaced by productivity logics

“*There are no emotional values in large farms. It is null. It has turned into an assembly line… and they are basically production values. Yes, of course, you do find hyper-acute mastitis in high-production animals. And she will end up with half the milk or die… that animal matters more to them*.” ES_VF.07

We also identified the differentiation between species to be embedded in the legal and policy frameworks that govern antimicrobial use across the countries investigated. For example the EU Veterinary Medicines Regulation (11) explicitly prioritizes certain species. This means from January 2024 EU is requiring all Member States to collect antimicrobial use data for cattle, pigs, chickens and turkeys. In January 2027 for all remaining food-producing animal species and only until January 2030 will be mandatory for other animals that are bred or kept (this includes dogs), with an explicit exemption

“*nothing… shall be understood to include an obligation to collect data from natural persons keeping companion animals*.” (EU 2019/6, Art. 57)

Similarly, Brazil's PAN-BR AGRO (22) programme was launched with the mandate to “*promote the rational use of antimicrobials in agriculture*” (Objective 6), focusing its early actions on production animals such as broilers (“*implement a surveillance programme of antimicrobial resistance in bacteria isolated from broiler chickens*”). Pets, by contrast, were left aside and only addressed later through a separate 2022 guide for dogs and cats (24).

### 3.2 Human reality and imagined animal needs

Another recurring theme was the discrepancy between what an animal might require and what it actually received, with care practices often shaped by imagination rather than confirmed need. Veterinarians described making decisions based on what they assumed tutors expected or what they felt legislation permitted, frequently without the benefit of a formalized diagnosis.

For example, this dynamic was evident in small animal practice, where dermatology specialists invested extensive time in examinations and communication

“*The difference is that I work without time limits. If it takes an hour to explain the causes or how they should apply the treatment, I do it without barriers. Also, there is a lot of communication, especially during the first days*.” ES_VF.09

Yet, client expectations for fast, cheap, and practical solutions led to quick prescribing in other cases. A dermatologist illustrated how expectations shaped compliance

“*If you say, ‘If you do this, there is a chance that we can avoid the use of antibiotics,' they try harder. Instead of saying they have to do it, then they want the pills instead*.” SE_S.05

Yet at times preconceptions and poor trust shapes the decisions

“*There will never be an animal that does not get infected in the kennel. We take every precaution during surgery, but the next day it will be in an environment that I don't trust, and I feel more comfortable if I give an antibiotic [post-surgery] to prevent infection.”* ES_VD.08

In dairy clinics, veterinarians often rushed to treatment

“*Ceftiofur is usually the first line used because of the issue that it has no withdrawal time in milk… because then the intramammary has withdrawal time, and the injectable, it doesn't, so they prefer to use injectable*.” BR_VF.06

except in cases where animals were perceived as having high value

“*Sometimes you initiate an antibiotic treatment, even though you know the prognosis might not be super good… If I understand from* [the farmer] *that it's a highly valuable cow, then I take it into account.”* SE_S.01

This assumption of the need to prescribe was not always abstract; sometimes, it was reinforced by tutors' demands for action

“*I'll tell you honestly, the producer wants us to solve the problem. And sometimes, if you go there and say, oh, let's not do it, he speaks badly of you, says you are wrong, you know, he doesn't give you credibility. So, sometimes… you end up under pressure to use it*.” BR_VF.07

Interestingly, when faced with this reality, veterinarians can recognize these biases, observable not only with colleagues but also their clients

“…* the farmers are very good, I believe in them, but sometimes their perceptions are biased too, right? Just like ours*.” BR_S.09

Legislation also functioned as a means to frame what can be done, with veterinarians sometimes frustrated that drugs available in other countries or species could not be used under current national rules, even when they believed such treatments would better serve the animal. The result was that antimicrobial use was often organized around professional and tutor perceptions rather than around systematically verified animal needs.

### 3.3 Who is the veterinarian? Who diagnoses and who supplies? Para-veterinary roles

Although legislation in all three countries reserves the acts of diagnosis and prescription for veterinarians, the practical reality in dairy farms and clinics revealed blurred professional boundaries. The blurred professional boundaries that shape who diagnoses and who treats, are themselves expressions of the hierarchy of care, showing how species value and practical constraints redistribute veterinary responsibility. The EU Regulation establishes that

“*a veterinary prescription shall be issued only after a clinical examination or any other proper assessment… by a veterinarian*.” (EU 2019/6, Art. 34–35) (11)

Spain follows these principles in its regulations:

“V*eterinary prescriptions will only be issued after a clinical examination by the prescribing veterinarian*.” (Real Decreto 666/2023, Art. 32–33) (18)

In Sweden, the Board of Agriculture requires that

“…* even when you issue a veterinary prescription… before issuing it you must have made a diagnosis*.” (34)

Brazil's federal professional law defines

“*the prescription of veterinary medicines…* [as] *a veterinary medical act considered to be the exclusive domain of veterinarians*.” (23)

Moreover, the Brazilian Ministry of Agriculture dictates under the “Responsible Use of Antimicrobials” guidance (27, 28) that

“*veterinarians must… prescribe antimicrobials based on clinical knowledge… supported by laboratory diagnostics…*.”

However, farmers, dog tutors, or even commercial intermediaries, who were not always veterinary professionals, often initiated diagnosis and treatment decisions. In Spain, large farms relied on protocols left by veterinarians

“*…you set up decision trees for people to follow. On these large farms, you give people the power to make decisions*.” ES_VF.07

In Brazil, veterinarians described how producers or farmers would directly administer antimicrobials

“*The problem is that he ends up calling the neighbour to solve it for him. Then the neighbour comes, gives a dose of antibiotics… So I think it's a lack of professionals giving support to farmers…*.” BR_VF.09

Such treatment decisions, organized around producers' perceptions rather than formal diagnosis, had consequences for antimicrobial stewardship, such as use of broad-spectrum antimicrobials, early cessation of treatment, and non-optimal use

“*They used to give antibiotics for two days until the cow got better. They saw that she improved, then they stopped treatment.” BR_VF.03*

“*Most use Kinetomax, right, Enrofloxacin. It's all over the place… a lot, a lot, a lot. I see that they also use a lot of ceftiofur*.” BR_VF.07

Another aspect described was the role of agricultural suppliers, where sales dynamics intersected with treatment decisions:

“…*the farmer goes to the agricultural store, says ‘my cow has this and that and such…' The guy is going to sell; he lives off sales. He is a veterinarian, but he doesn't know the farm, he doesn't know the cow, doesn't know the management, and he's going off the farmer's perception, but sometimes his perception… So, the sale of antibiotics is really high, right? It makes money, this makes a lot of money*.” BR_VF.10

In Sweden, delegation was visible in dairy reproduction consultations, as explained by an expert:

“*Artificial insemination technicians are allowed to do diagnosis, but not treatments… Farmers… they can diagnose fertility disorders, but then they have to ask a veterinarian if treatment is needed … can inseminate their own cows but not do pregnancy checks or treatments*.” SE_S.03

Delegation of veterinary duties is also shaped by technology access, questioning the need of a veterinarian to do a diagnosis.

“*… our presence on the farm is decreasing. Reproduction now is nothing like it used to be. It's a matter of looking at the Herd Navigator [on-site diagnostic tool] and seeing four cows with ovarian pathology. The rest is done by the computer [software]… I see that technology is there to do all this. We veterinary diagnosticians are becoming obsolete. There will be machines that do it better.”* ES_VF.17

This distribution of responsibility blurred the boundary of diagnosis. Veterinarians themselves recognized the dynamic

“*Dairy farmers, they are good. So, they pretty much phone you because they have this disease, and you just have to confirm that. They rarely phone you because of something strange or bizarre that they don't understand. So, in most cases, they know what the problem is. And they know the treatment as well*.” SE_S.02

“*Dairy farmers know as much about mastitis as any veterinary graduate, if not more….”* ES_FV.03

Farmers expressed a similar view:

“*We see that it is a mastitis, we know what it is. But then we bring in the veterinarian to get the medicine, so to say*.” SE_DF.04

Small animal practice showed parallel tendencies. For example, in Brazil, pet veterinarians pointed out the easy availability of antimicrobials without prescription or the prevalence of prior treatments ahead of consultations

“*I think the main thing is the purchase, right? At agricultural stores, at pet shops, and without a prescription. They don't need a veterinarian's diagnosis or prescription, so it's just showing up*.” BR_VD.08

“*…it's always like that, you know, because people buy [medicine] at agricultural stores. The animal shows up, and it's already had a bunch of stuff….”* BR_VD.14

These dynamics, also evident in small animal and large animal practice, where dog tutors sometimes pursue “quick fixes,” illustrate how responsibility for AMU is distributed across multiple actors, with veterinary authority diluted in everyday care.

### 3.4 The focus on numbers, not context—follow-up: is no news good news?

Follow-up emerged as a fragile and inconsistent element of antimicrobial use practice. In this context, follow-up refers to the process of checking whether a prescribed treatment has achieved its intended effect, which requires documenting the rationale for prescribing, the course of treatment administered, and its success or failure.

Follow-up was left to informal practices

“*I think the follow-up has been a little deficient, really! It has been a bit like ‘get in touch if it doesn't get better'… if they don't get in touch, then it is good and then I have judged that then this treatment went okay, kind of!”* SE_VS.07

and outcomes were seldom documented

“*We don't measure success of treatment systematically. Generally, we don't take note of that.”* SE_S.02

Veterinarians rarely received feedback about treatment success

“*I ask for a call back the next morning, but rarely is there a final call after five days to see if the animal recovered.”* SE_S.02

Participants' experiences seemed tied by the logic of “no news is good news,” the assumption that absence of feedback was a sign of recovery

“*…if I don't hear anything after a couple of days, I assume it worked. But of course, it can also mean that the cow died or that they called another vet. We don't really get that feedback systematically.”* SE_S.01

Veterinarians pointed out the inefficiency of this informal and incomplete process *for example*,

“*… and what happens a lot too, you know, is… it comes to the vet saying “I know this works”… but there's no real follow-up….”* BR_VD.14

“*For follow-up, … you trust the farmer to call, because you know that his perception will be the real one.”* ES_VF.03

Moreover, participants described how follow-up was shaped differently according to species. In dairy farming, follow-up was often linked to herd-level monitoring rather than individual animals

“*Not every epidemiological unit is useful, you can't focus on the individual, it's the farm as a whole that you manage… Every four months, we send a number of mastitis samples for an antibiogram and a culture to better monitor the farm.”* ES_VF.07

In companion animals, expectations of closer feedback appeared sometimes

“*… one or another, up until today, I still get news about. Sometimes they send me, ‘Hey, look how it's doing, it's like this and that.' But most of them… they end up being breeders, and soon those puppies are going to be sold… I completely lose contact.” BR_VD.06*

Due to the caretaker context (assumed or real), the imagined animals' needs further muddle treatment follow-up

“…* follow-up also depends on the dog tutor…. You can prescribe something, but the carer will try something else on their own. This is the case of an overzealous carer who will use their own remedies. Those of us [veterinarians] in rural areas know that they are not going to have good hygiene, so we compensate for the lack of hygiene at home… If we doubt compliance, we give them injections [antibiotics] to ensure they receive the treatment.”* ES_VD.09

Other reflections link follow-up to structural features of farming. In Spain, herd size and farm management influenced how treatments were continued and supervised

“*The larger the farm, the poorer the milking practices tend to be… We're not the ones who start or finish all of the clinical treatments.”* ES_VF.07

“*…by having two or three visits a week scheduled at those large farms, the cow can wait for you, or the farmer can just give you a call.”* ES_VF.08

In Brazil, concerns centered on the weakness of records

“*We can't really assess a situation if we don't have a trustworthy record, you know*?” BR_S.09

and the effect of self-medication on evaluation of treatment success and record keeping

“*…the producer will perform this treatment* [without veterinary consultation]. *If he does not know what the active ingredient is… he may not solve the problem… in his mind, he solved the problem, but in reality, the problem is still there, right? It often becomes subclinical mastitis again because he did not treat the problem….”* BR_S.05

Legislative frameworks structure follow-up differently according to species. At the international level, the WHO frames veterinary antimicrobial stewardship largely around livestock, stating that “responsible use should reduce or restrict the use of antimicrobials in food animals” (46). At EU level, Regulation (EU) 2019/6 (11) required Member States to collect antimicrobial use data first for all food-producing animal species. The Regulation also specifies that there is no “*obligation to collect data from natural persons keeping companion animals”* (11). This means that while phased reporting is mandatory for livestock and other kept species, there is no obligation to capture usage data directly from pet tutors, contrary to what is demanded from farms. Spain followed European regulation through the Royal Decree 666/2023, establishing obligatory treatment records for production holdings, linking them to the EU system (18). In January 2025, Spain extended mandatory electronic prescriptions to include dogs, introducing requirements such as animal identification; the system's poor fit with small-animal clinical practice led to disputes and forced special dispensations in the legislation (17). On the other hand, Sweden has a long history of annual national reports on antimicrobial use and antimicrobial resistance in human and sectors. Yet, initially, these reports focused on livestock species such as poultry, pigs, and cattle, with other animals added later. As the Swedish Veterinary Institute notes:

“*Sales of antibiotics for animals has decreased by around 70 percent since the mid 1980-ies … the key strategy has been prevention of disease—healthy animals do not need antibiotics*.”(40)

In Brazil, the PAN-BR AGRO plan concentrated its initial surveillance on production animals, particularly pigs and poultry, while companion animals and most dairy contexts were omitted (22). Strict prescription documentation was mandated only for a subset of controlled substances under Instrução Normativa n° 35/2017, resulting in many antimicrobials not being systematically tracked (29).

### 3.5 Treatment-first logics

Across geographies and species, veterinary practice appeared consistently organized around a treatment-first logic, where veterinary diagnostic reflection was frequently bypassed, and antimicrobials became the default means of responding to animal illness. This pattern was not only a matter of convenience but reflected broader social and professional meanings, including the urgency to resolve problems for valued animals, the framing of guidelines as therapeutic lists, and the material conditions of practice.

Legislation and professional frameworks agree that antimicrobial use requires a prescription, and a prescription requires a diagnosis by an accredited veterinary professional (11, 18, 23, 28, 33, 36). In Spain, stewardship was framed by prescribing guides structured as lists of antimicrobials tailored to particular conditions, rather than by diagnostic algorithms; they prioritized selecting the “correct” drug over encouraging diagnostic reasoning (20). In Brazil, regulations mandate strict documentation only for a subset of veterinary substances under special control. For instance, opioids such as fentanyl are on the MAPA special-control list and require a formal prescription via the *Notificação de Receita Veterinária* system (30). In contrast, widely used antimicrobials like amoxicillin, a critically important drug for both human and veterinary use, fall outside that requirement and remain less systematically tracked (29). In Sweden, the Veterinary Association introduced guidelines for the clinical use of antimicrobials in dogs, cats, and livestock (38, 39). These guidelines provide condition-based recommendations, emphasizing the need to avoid antimicrobial treatments whenever feasible and encouraging non-antimicrobial options based on formal diagnosis.

Yet, even in Sweden, farmers often identify health problems themselves, calling veterinarians mainly to supply medicines

“*… they will not call a vet, not until the cow gets really sick. Because they have been taught that it should be a conservative treatment of those cases.”* SE_S.03

blurring the diagnosis process. Spain and Brazil followed similar patterns. For example, in small animal practices in Brazil, animals that arrive at clinics often have already been medicated

“*It is rare to receive a sample that has not already been treated with antibiotics before performing the culture and antibiogram test…*” BR_VD.05

with antimicrobials given at home or purchased directly from agrovet shops

“*The treatment I base myself on is whether I can get this or that medicine. The drugs have already been bought. They are not prescribed, no! That's how it is. Some vets sign and so on, but the medicine has already been bought. If they call and bring me Ceftiofur, I will use Ceftiofur.”* ES_VF.17

When antimicrobials were prescribed, they were sometimes trialed sequentially or given at incorrect doses and durations, reflecting a search for rapid improvement rather than systematic diagnosis

“*I understand that many times we can't keep up, or for those who are clinicians, they no longer remember much from microbiology. But sometimes they try one, then another, then another, and they don't stop to think, they don't diagnose, they don't know which microorganism they are dealing with, and then they just keep testing until something works clinically. That's how I see it. The main issue for me is the ease of purchase and then the way veterinarians themselves use the drugs. This isn't only in small-animal practice, you know….”* BR_VD.08

In dairy practice, farmers pressed for immediate solutions

“*My dream is to have my own antibiogram… I don't feel confident enough to make a correct diagnosis… Producers want you to solve their problems right away 'My cow has mastitis right now, and I want her to get better by tomorrow.”* BR_VF.03

and veterinarians described resorting to broad-spectrum antimicrobials for prevention, bypassing formal diagnosis

“*The ideal is this, you apply antibiotics only to those who need it… but if the farm does not have good conditions… it is more advisable to use dry cow therapy and a teat sealant on every cow*.” BR_VF.08

Participants' experiences highlight antimicrobials as a primary tool of care, at times reducing the veterinary role as a provider of antimicrobials

“*Veterinarians are really just a way to shoot antibiotics.” SE_F.03*

In others, reforms and policy changes have reduced inappropriate prescribing

“*When I was young… in the beginning of the eighties … we used a lot of antibiotics… we even had dogs on low-dose preventative antibiotics. Then the national policy document came in 2007, and within two years, prescriptions really dropped.”* SE_S.05

but treatment-first routines persisted, sustained by professional habit and expectations “*Some colleagues* [refering to veterinarinas] *you just can't reach…”*. In life-threatening situations, using antimicrobials was framed as ethically unavoidable, regardless of diagnostic uncertainty. Described patterns reaffirm how a “hierarchy of care” shapes daily decision-making, where species value and structural pressures converge to sustain rapid, treatment-first routines.

## 4 Discussion

**Figure 2:**
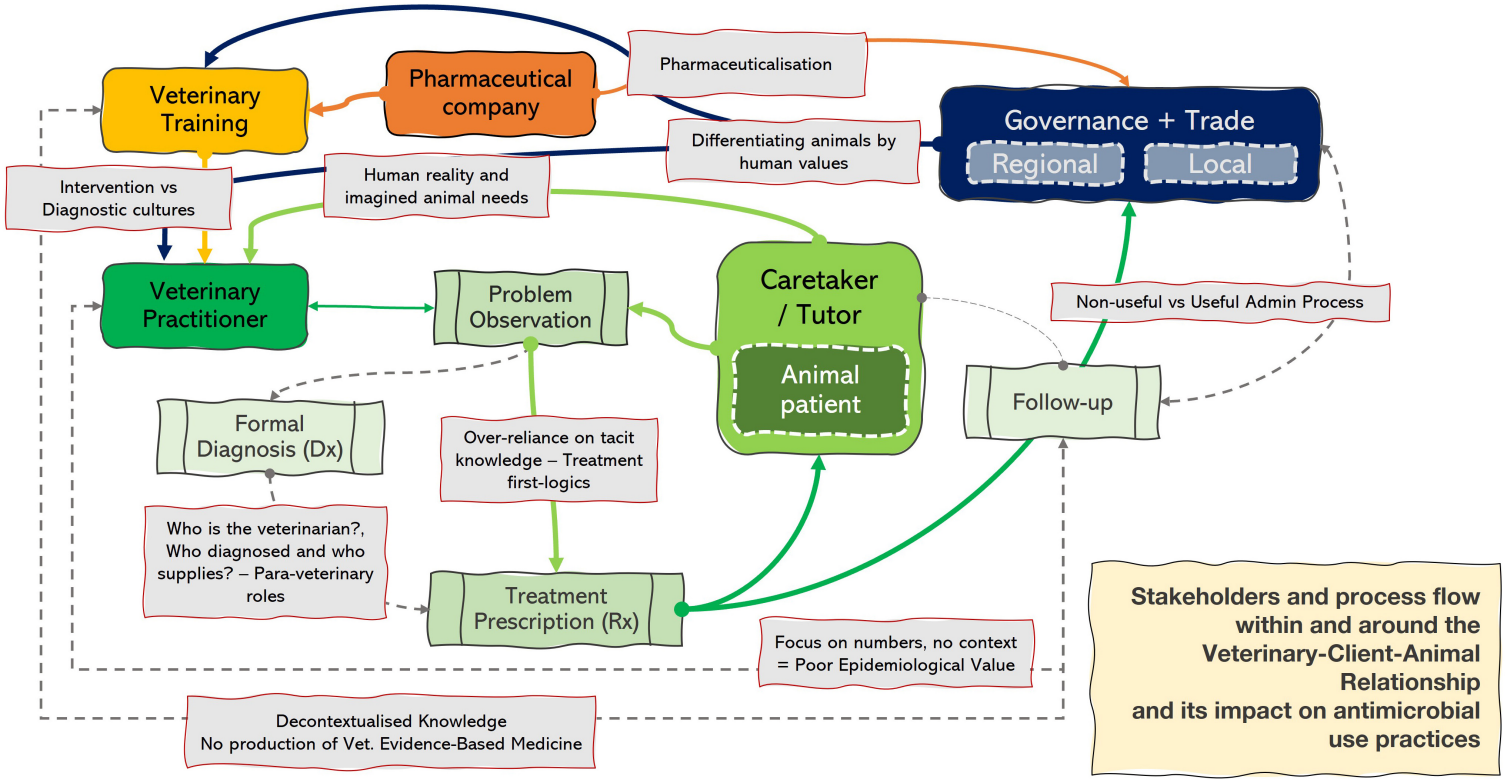
Diagram illustrating how antimicrobial use is shaped within the Veterinary-Client-Animal relationship. Solid arrows indicate active connections; dashed and gray lines mark weakened or missing links. The central pathway (problem observation → diagnosis → treatment → follow-up) highlights where decision-making breaks down, reinforcing treatment-first logics and weak feedback loops. A key finding is the frequent bypass of formal diagnosis, with veterinarians moving directly from problem observation to treatment. This is reinforced by pharmaceuticalisation, client expectations (imagined animal needs), and a sustained culture of intervention, treatment-first logics. Fragmented or weak follow-up prevents contextualized learning, undermining evidence-based medicine and limiting opportunities to refine stewardship in practice.

Based on Shove's Social Practice Theory (7, 8) and emphasizing the element of meanings, this study used reflexive thematic analysis to identify interconnected themes that describe how antimicrobial use is enacted in veterinary practice across species and geographical contexts (Figure 2). Antimicrobial use emerged not as a purely technical act governed by clinical guidelines, but as a practice shaped by the social significance veterinarians and caretakers attributed to different animals, added to their assumptions of what care and antimicrobial use should entail, and a diffuse re-distribution of what ought to be veterinary professionals' only responsibilities. Limited systems of follow-up and record-keeping further marked these practices, along with a treatment-first logic culture, in which antimicrobial treatment often became the taken-for-granted response to a problem observation. Together, these themes articulate what we interpret as a hierarchy of care in veterinary medicine, where the meanings attached to species underpin both tolerance for antimicrobial use and lack of tailored treatment aligned with actual animal needs.

We show how the value assigned to different species and the imagined needs that guide decisions shape veterinary antimicrobial practices. Such findings highlight the role of speciesism in shaping veterinary antimicrobial practices. Here, we define speciesism as the unjustified, disadvantageous consideration or treatment of those not classified as belonging to a particular species (47). Speciesism has been discussed in other contexts (48–50). Still, our analysis is novel in showing how it operates within veterinary medicine, specifically within the practice of antimicrobial use, to reinforce hierarchies of care, privileging some animals over others based on their perceived social or economic value. Animals perceived as having high value were often fast-tracked into treatment with broad-spectrum or critically important antibiotics; in contrast, lower-valued animals, typically livestock, were usually managed in suboptimal standardized protocols that obscured individual needs. In both cases, tailored optimal antimicrobial use was compromised. Weak reporting systems meant that these practices rarely generated learning or accountability. These dynamics illustrate how species-based valuation contributes to poor veterinary antimicrobial stewardship, reinforcing pathways that accelerate AMR while shaping animal welfare outcomes.

This focus also reveals an economic hierarchy within food-producing animals. In Brazil, poultry have been prioritized over other industries, including dairy, because of their high economic value and importance for export markets (22, 51, 52). In Spain, the success of national AMR programmes has been measured largely through progress in a single species, the swine sector, showing how stewardship outcomes are often tied to sectors of most significant economic weight rather than to a balanced approach across production systems (14–16, 53). Such differentiation demonstrates how affective vs. productive valorisation structures shape antimicrobial practice, with regulatory frameworks and stewardship programmes reproducing these hierarchies rather than challenging them.

Another critical aspect concerns the observed pharmaceuticalisation of animal health problems and the expectation that veterinarians should exercise a formalized diagnostic process before prescribing antimicrobials. This does not simply mean carrying out laboratory tests, but rather exercising what Kahneman has described as “*slow thinking”* (54), *a deliberate, reflective process of considering what* the diagnosis actually is, what needs to be treated, and only then deciding on the appropriate therapeutic pathway (55, 56). Our findings suggest that, in many cases, this reflective diagnostic stage was bypassed. The absence of a diagnostic reasoning reinforced a culture of treatment-first practice, diminishing opportunities for targeted and optimal antimicrobial use and further entrenching patterns of pharmaceuticalisation. This emerging concept refers to a socio-technical process where medical conditions, capabilities, or needs are increasingly framed as opportunities for pharmaceutical intervention (57). When failure was not an option for high-valued or emotionally significant animals, treatment was fast-tracked, while lower-valued animals were managed through standardized shortcuts. In this way, pharmaceuticalisation intersected directly with speciesism and with imagined valuations of animals, linking together several observations.

The treatment-first culture revealed in our study is not merely a failure of compliance but situated practices produced by the interaction of infrastructures, competences, and the meanings attached to animals. Also, these logics are not uniform but take different forms depending on geography, species, and infrastructure. For example, in Brazil and Spain, over-the-counter availability of drugs and weak documentation requirements mean that animals often reach the clinic already treated, reducing diagnostic value. Preventive blanket use in dry cow therapy was rationalized as unavoidable when farm conditions made selective use impractical. More nuanced, the practice guidelines for optimizing antimicrobial use in Spain focus on therapeutic lists rather than frameworks for differential reasoning (20). Such regulatory tools implicitly shift attention from the question of whether to use antimicrobials to which antimicrobial to choose. In Sweden, diagnostic confirmation was more embedded in training and practice. Yet, students described how decisions still varied by context and animal value. The preventive use of penicillin for high-value cases illustrated how treatment-first logics persist even in contexts with stronger stewardship traditions. This shows that cultural meanings attached to animals can override regulatory ideals.

Across all three countries, the professional boundary of “only veterinarians can diagnose and prescribe,” clearly stated in the national and international legislations (11, 18, 23, 28, 33, 36), was consistently blurred. Brazil represented one extreme, where farmers, neighbors, or commercial intermediaries often made treatment decisions. Although veterinary confirmation was formally required in Sweden and Spain, it was frequently delegated or assumed. Such blurring does not always equate to excessive antimicrobial use, but it shows that veterinary practice is co-produced with farmers, technicians, and other actors. Stewardship efforts must look beyond veterinarians and engage the wider networks that shape animal health decisions. Our findings underline the value of examining the broader systems veterinarians operate in, as we have done in this project. Focusing only on individual prescribing habits would give an incomplete picture, while situating practices within their social, economic, and institutional contexts reveals how antimicrobial use is produced and sustained.

The absence of systematic follow-up and outcome recording further undermined learning and stewardship. While prescriptions were logged, information on why a drug was prescribed or whether it worked was rarely captured. As a result, current monitoring frameworks risk reducing stewardship to a quantitative exercise, centered on counting doses without reporting appropriateness or outcomes. This process erodes the principles of evidence-based medicine (58, 59), from which the veterinary profession derives its capacity for improvement. Efforts to optimize antimicrobial use will remain constrained without embedding follow-up and outcome recording.

An important dimension running through all the themes is imagination: how animals are imagined in terms of their value, how their needs are imagined in practice, and how treatments are imagined in light of legislation and client expectations. Often, decisions were shaped more by these assumptions than by systematic diagnostics. Although imagination may enable fast thinking (54, 56), it undermines stewardship when choices prioritize the preferences of clients, employers, colleagues, or even professional self-satisfaction over the actual needs of the animal. Addressing this requires improving communication with stakeholders and explicitly recognizing conflicts of interest. Yet our findings also show that these tensions are rarely discussed openly in veterinary training. Students were acutely aware of how species were valued and questioned why mentors handled them differently. Still, this critical appraisal seemed to diminish once they entered practice, where legal frameworks, farm infrastructures, and client demands became overriding pressures. Opening space for dialogue, beginning in training and continuing throughout professional life, would enable veterinarians to critically appraise their clinical decisions and the human values embedded in them. Balancing those values with the needs of animals themselves would foster more optimal use of antimicrobials, reduce the risk of resistance, and enhance professional satisfaction.

## 5 Conclusion

This study draws on social practice theory to demonstrate the complexity of decision-making and the role of context underlying veterinary practice relating to AMU. Veterinarians do not work in isolation; they are strongly influenced by their inherent assumptions of client expectations and animal value, as well as the values of the system in which they operate. We can only appreciate the nuances surrounding veterinary antimicrobial practice in different geographical contexts and species by considering the wider system. Our findings highlight the hierarchy of care within veterinary practice that drives decision-making and treatment choice depending on the perceived value of the animal concerned, ultimately impacting antimicrobial use and animal welfare. The adoption of a treatment-first, rather than diagnostic-led approach, has significant implications for responsible antimicrobial use, stewardship and public health, and we strongly recommend that this is addressed within the veterinary context. Although the study focused on Brazil, Spain, and Sweden, many patterns identified, such as blurred professional boundaries, weak follow-up, and treatment-first logics, are shaped by structural rather than economic factors. Similar dynamics are likely to occur across and within countries, suggesting stewardship efforts should focus on improving diagnostic support, communication systems, and feedback mechanisms wherever these structural gaps persist.

## Data Availability

Due to privacy considerations, full interview transcripts will not be shared, as many participants come from a small community and may risk identification. However, the corresponding author can summarize relevant interview quotes upon reasonable request. Requests to access the datasets should be directed to Dr. Gabriela Olmos Antillón, gabriela.olmos.antillon@slu.se, gabriela.olmosantillon@gmail.com.
